# Effect of Tamarind Seed Polysaccharide on the Quality Characteristics and In Vitro Digestibility of Frozen Steamed Buns

**DOI:** 10.3390/gels12060461

**Published:** 2026-05-25

**Authors:** Xingmei Sheng, Qi Cui, Siyan Huang, Zibo Song, Xueming Xu, Junjie Yi, Chaofan Guo, Yongshuai Ma

**Affiliations:** 1 Faculty of Food Science and Engineering, Kunming University of Science and Technology, Kunming 650500, China; sxm00128@163.com (X.S.); cuiqi1216@163.com (Q.C.); junjieyi@kust.edu.cn (J.Y.); guochaofanfan@outlook.com (C.G.); 2 Yunnan Key Laboratory of Plateau Food Advanced Manufacturing, Kunming 650500, China; 3 Yunnan International Joint Laboratory of Green Food Processing, Kunming 650500, China; 4 Yunnan Maoduoli Group Food Co., Ltd., Yuxi 653100, China; huangsiyan@maodudi.com (S.H.); 1354630803@qq.com (Z.S.); 5 Yunnan Special Favor Biotechnology Co., Ltd., Yuxi 653100, China; 6 Yunnan Provincial Key Laboratory of Applied Technology for Special Forest Fruits, Yuxi 653100, China; 7 School of Food Science and Technology, Jiangnan University, Wuxi 214122, China; xmxu@jiangnan.edu.cn

**Keywords:** tamarind seed polysaccharides, resistant starch, water binding capacity, freezing stability

## Abstract

This study evaluated the effects of tamarind seed polysaccharides (TSP) on the quality characteristics and in vitro starch digestibility of steamed buns made from doughs with different freezing storage times (0, 30, and 60 days). The pore structure, specific volume, water distribution, and starch digestibility were analyzed. TSP significantly altered the dough microstructure by increasing pore density and pore volume while reducing the average pore area, forming a more uniform pore network. During freezing storage, the specific volume of control samples decreased, whereas steamed buns with 1–2% TSP maintained a relatively high specific volume (~1.65) after 60 days, indicating improved gas retention and structural stability. TSP also increased bound water and restricted water migration. Additionally, TSP increased resistant starch (RS) from 15.96% to 24% and reduced rapidly digestible starch (RDS). Overall, TSP improved the structural stability of frozen steamed buns by regulating water distribution, strengthening the gluten-starch network, and altering starch digestibility. These findings provide insights into the use of natural polysaccharides to enhance the quality and nutritional function of frozen wheat-based foods.

## 1. Introduction

Wheat is one of the most widely cultivated cereal crops worldwide and serves as a primary raw material for numerous staple foods. The functional properties of wheat flour are largely determined by the interactions among its major components, including starch, gluten proteins, and non-starch polysaccharides, which collectively govern dough rheology, gel formation, and the structural characteristics of final products [1,2]. Among wheat-based foods, steamed buns are widely consumed in many Asian countries due to their soft texture, uniform crumb structure, and convenient preparation. During dough processing and steaming, starch gelatinization and gluten gel network formation occur simultaneously, forming a complex gel microstructure that determines key quality attributes such as pore distribution, specific volume, and crumb softness [3,4]. With the rapid development of convenience foods, frozen steamed buns and frozen dough products have gained increasing industrial attention because of their extended shelf life and ease of transportation and storage. However, the freezing process often leads to undesirable physicochemical changes in wheat-based products. Ice crystal formation and recrystallization during frozen storage can disrupt the gluten gel network and damage starch granules, resulting in moisture redistribution, crumb firming, and structural collapse [5]. Furthermore, repeated freezing and thawing cycles may accelerate starch retrogradation and alter the thermal and rheological behavior of starch, thereby negatively affecting the texture and gel structural stability of wheat-based foods [6]. These structural deteriorations ultimately reduce product quality and consumer acceptance. Therefore, developing effective strategies to regulate water migration and maintain the gel structural stability of frozen wheat-based foods has become an important research focus.

Hydrocolloids are widely applied in flour-based systems because of their ability to regulate water mobility, modify dough rheology, and stabilize food gel microstructures. Through gel-forming interactions with starch granules, gluten proteins, and water molecules, hydrocolloids can enhance gas retention during fermentation, strengthen crumb structure, and delay staling during storage [7]. The studies by Ashraf et al. [8] and Liu et al. [9] demonstrated that hydrogels have a significant impact on the physical properties and microstructure of bread based on wheat and frozen baked foods, among others. Moreover, hydrogels can change the digestibility of starch by altering the structural organization of the starch matrix or forming physical barriers that restrict enzymatic hydrolysis. Tamarind seed polysaccharide (TSP) is a naturally derived polysaccharide extracted from *tamarind seeds*, which has attracted increasing attention as a functional gel-forming hydrocolloid in food systems [10]. Structurally, TSP consists of a β-D-(1→4)-glucan backbone substituted with arabinose and galactose side chains, forming a branched polysaccharide gel network with strong intermolecular interactions [11,12]. This structural configuration endows TSP with excellent thickening ability, strong water-binding capacity, and remarkable resistance to thermal and mechanical stresses [13]. Xie et al. [14] discovered that TSP significantly alters the gelatinization behavior, rheological properties, and gel microstructure of starch systems. Moreover, adding TSP can improve the texture and structural stability of gluten-free cakes and significantly enhance the physical and chemical properties of starch-based systems. TSP may be an effective component for improving the gel structural stability and functional properties of cereal-based foods [15].

Although studies have shown that TSP can improve the starch system and food structure, its mechanism of action in complex wheat flour gel systems and quality regulation of frozen steamed buns remains unclear [16,17]. In particular, the research on the mechanism of TSP’s influence on the dough structure development, water migration behavior within the gel matrix, and starch digestibility of steamed buns during freezing storage needs to be further explored. Understanding these interactions is crucial for clarifying the structure-function relationship of polysaccharide gels in cereal-based systems and improving the quality stability of frozen wheat products.

Therefore, the objective of this study was to investigate the effects of TSP on the quality characteristics and in vitro starch digestibility of steamed buns during frozen storage. Steamed buns containing different concentrations of TSP were prepared, and their gel pore structure, specific volume, color attributes, and texture properties were systematically evaluated. The internal water migration within the gel network and the digestion characteristics of the dough were analyzed to clarify the structural and functional roles of the gel system in the wheat flour system and to provide a theoretical basis for the application of natural polysaccharides in improving the quality and nutritional functions of frozen wheat-based foods.

## 2. Results and Discussion

### 2.1. Pore Structure Analysis of Steamed Buns

Porosity, average pore area, and pore density are key indicators for evaluating the uniformity of internal structure and the integrity of the network in baked products prepared from frozen dough. In frozen dough systems, ice crystal growth, disruption of the gluten network, and water migration can significantly affect gas retention and pore stability. Therefore, these parameters can be used to assess the extent of structural deterioration during frozen storage and the protective effect of TSP [18].

**Figure 1:**
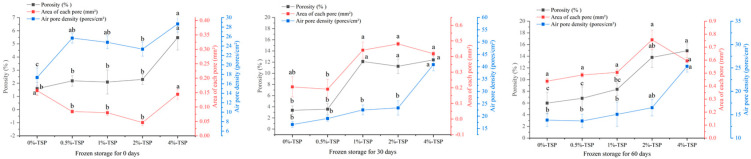
Effects of TSP addition and frozen storage time on pore structure parameters of *steamed buns* prepared from frozen dough. The concentrations labeled as 0%, 0.5%, 1%, 2%, and 4% correspond to 0, 0.5, 1, 2, and 4 g/100 g (*w*/*w*, based on wheat flour), respectively. Different letters were significantly different (*p* < 0.05).

As shown in Figure 1, at 0 d of frozen storage, the porosity and pore density increased significantly with increasing TSP addition, while the average pore area decreased, indicating that TSP promoted the formation of a pore structure with a greater number of smaller and more uniformly distributed pores. As the frozen storage time extended to 30 and 60 d, the porosity and pore density of all samples increased markedly, accompanied by a significant enlargement of the average pore area. This result suggested that ice crystal growth and recrystallization during frozen storage disrupted the gluten network, weakened gas retention capacity, and led to pore coalescence and structural coarsening. However, compared with the control group, samples containing TSP maintained higher pore density and relatively smaller average pore areas at all freezing stages, particularly at higher addition levels. This indicated that TSP effectively stabilized the pore structure by enhancing water-holding capacity, improving dough viscoelasticity, and inhibiting network damage caused by ice recrystallization. Overall, TSP significantly improved the stability of the pore structure of dough products during long-term frozen storage by strengthening the gluten network and regulating water distribution.

### 2.2. Specific Volume of Steamed Buns

The specific volume of steamed buns is an important indicator for evaluating volume expansion and internal gas cell formation. It reflects the integrity of the gluten network and its gas-holding capacity during fermentation and steaming, thereby comprehensively representing the ability of frozen dough to generate, retain, and stabilize CO_2_ during processing [19].

**Figure 2:**
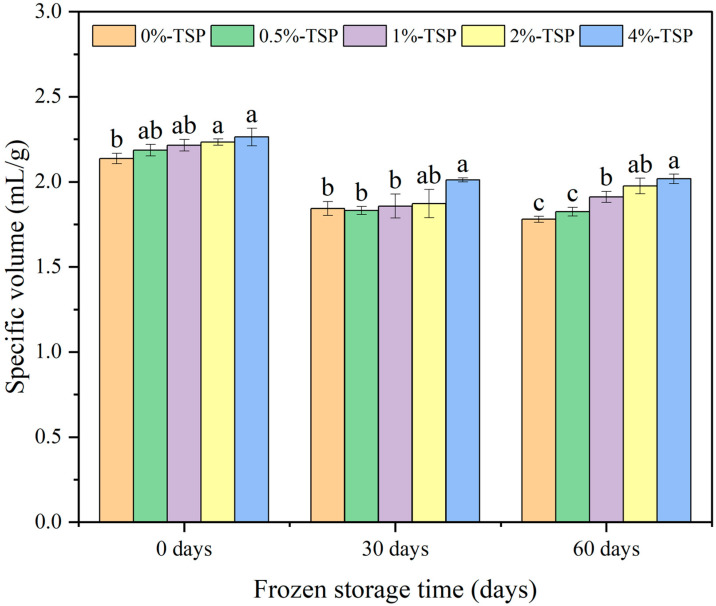
Effects of TSP addition and frozen storage time on the specific volume of *steamed buns* prepared from frozen dough. The concentrations labeled as 0%, 0.5%, 1%, 2%, and 4% correspond to 0, 0.5, 1, 2, and 4 g/100 g (*w*/*w*, based on wheat flour), respectively. Different letters were significantly different (*p* < 0.05).

As the frozen storage time increased from 0 to 60 d, the specific volume of the control group generally showed a decreasing trend, indicating that long-term freezing weakened the gas retention capacity and structural stability of the dough (Figure 2). This phenomenon was mainly attributed to the mechanical damage to the gluten network caused by ice crystal formation and recrystallization during frozen storage, while water migration reduced the continuity of the protein network, thereby lowering dough expansion capacity and product volume [20,21]. After the addition of TSP, this decreasing trend was significantly alleviated. In particular, samples containing 1–2% TSP maintained relatively high specific volumes (approximately 1.65) even after 60 d of frozen storage, indicating that an appropriate amount of TSP could enhance water-binding capacity, inhibit ice crystal growth and recrystallization, and promote polysaccharide-protein interactions to form a more stable composite network structure. These effects improved the gas-holding capacity of dough and delayed quality deterioration during frozen storage [22]. In contrast, the protective effect of 0.5% TSP was limited, possibly because the concentration was insufficient to form a continuous hydrated network capable of effectively suppressing ice-crystal-induced damage. Interestingly, the specific volume of the 4% TSP group showed a certain degree of recovery after 60 d of frozen storage (from 1.80 to 1.95). This phenomenon might be related to the gradual formation of a more continuous and stable hydrophilic gel network at high polysaccharide concentrations during prolonged freezing, which enhanced bubble film strength and structural recovery ability. However, this assumption requires further verification through rheological and microstructural analyses. Overall, an appropriate addition level of TSP (1–2%) effectively mitigated structural damage during frozen storage and exhibited superior long-term stability in maintaining the specific volume of steamed buns.

### 2.3. Moisture Distribution Analysis in Steamed Buns

Low-field nuclear magnetic resonance (LF-NMR) T_2_ relaxation spectra allow quantitative differentiation of water fractions with different mobilities. In this study, A21, A22, and A23 represented tightly bound water, moderately mobile immobilized/non-flowable water, and mobile pore/capillary water, respectively, and variations in peak area ratios reflected the distribution and mobility of water within the matrix [23].

**Figure 3:**
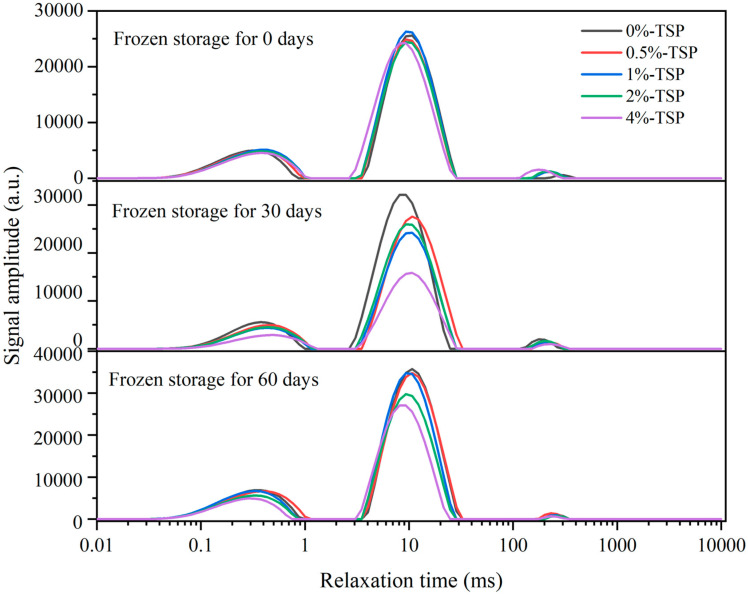
Effects of TSP addition and frozen storage time on relaxation time of *steamed buns* prepared from frozen dough. The concentrations labeled as 0%, 0.5%, 1%, 2%, and 4% correspond to 0, 0.5, 1, 2, and 4 g/100 g (*w*/*w*, based on wheat flour), respectively.

As shown in Figure 3, steamed buns produced from dough subjected to different freezing durations (0, 30, and 60 d) exhibited similar T_2_ spectral patterns. The spectra were dominated by a main peak around 10 ms, indicating that water in the final product primarily existed as restricted water confined within the three-dimensional network formed by starch gelatinization and protein denaturation during steaming. Smaller peaks within 0.1–1 ms corresponded to tightly bound water, represented by A21, whereas the minor long-T_2_ component at approximately 100 ms was assigned to A23, indicating the presence of mobile water within the pore or capillary structure. Compared with the frozen dough stage, the reappearance of the long-T_2_ component after thawing, proofing, and steaming indicated that ice-phase water reverted to the liquid state and redistributed within the porous crumb structure, placing part of the water in relatively free microenvironments detectable by LF-NMR. TSP addition markedly regulated the water state within the system. TSP addition altered the relative distribution of water populations, indicating that the polysaccharide regulated water binding and mobility within the matrix rather than producing a strictly dose-dependent decrease in free water. This behavior suggested that polysaccharides enriched hydrophilic groups capable of binding water through hydrogen bonding and increased system viscosity, thereby forming a composite network that restricted water migration toward the pore phase [24]. Quantitative analysis (Table 1) further confirmed that at 0 d of frozen storage, A22 was the dominant component in all groups, indicating that water mainly existed as restricted water after steaming, while A21 accounted for 19.50–21.03% and A23 showed the lowest proportion. The 4%-TSP group exhibited a significant decrease in A21 and a significant increase in A23 (*p* < 0.05), indicating that higher TSP levels altered the initial water distribution. After 30 d of frozen storage, the control group showed a marked decrease in A21, accompanied by increases in A22 and A23, suggesting that freezing-induced ice crystal formation and recrystallization promoted water migration and partially damaged the network structure. In contrast, TSP-treated samples showed smaller reductions in A21 and relatively lower A23 values, indicating a buffering effect of polysaccharides on water redistribution. After 60 d of freezing, A21 increased again in all groups, while A23 significantly decreased to 0.77–1.47%, which may be attributed to the hydroxyl-rich structure of TSP enhancing water binding through hydrogen bonding and forming a stabilizing composite network. Overall, prolonged freezing promoted ice recrystallization and water migration, generating microstructural defects in the dough network and affecting moisture redistribution in the final product, whereas TSP incorporation partially mitigated freezing damage and stabilized water distribution in steamed buns prepared from dough subjected to different freezing stages.

### 2.4. Textural Characteristic Analysis of Steamed Buns

Texture properties quantitatively reflect the internal network structure and sensory characteristics of steamed buns. Among these parameters, hardness and chewiness mainly represent structural densification and resistance to deformation, whereas springiness, cohesiveness, and resilience reflect structural continuity and reversible deformation ability.

The TPA results (Table 2) indicate that freezing storage significantly affected the texture of the steamed buns. As the storage time increased from 0 to 60 d, the hardness and chewiness of the samples generally increased, suggesting that frozen storage gradually induced a harder and more compact crumb structure. Meanwhile, springiness, cohesiveness, and resilience exhibited a decreasing trend during prolonged frozen storage, particularly after 60 d, indicating that the texture shifted toward a harder structure with reduced elasticity and weakened structural integrity. At the same freezing duration, the addition of TSP generally increased hardness, showing a dose-dependent trend. This phenomenon suggested that the water-binding, thickening, and filling effects of polysaccharides promoted the formation of a more compact network after steaming. However, after 30 and 60 d of frozen storage, although hardness increased with higher TSP addition, chewiness decreased with increasing TSP levels, accompanied by gradual reductions in springiness, cohesiveness, and resilience. These results indicated that excessive TSP might induce a hardening effect during long-term frozen storage while weakening structural continuity and reversible deformation capacity, resulting in a firmer mouthfeel. During frozen storage, the formation and recrystallization of ice crystals caused physical disruption of the gluten network and induced water migration, thereby weakening bubble films and the network skeleton. After thawing, proofing, and steaming, the damaged network tended to reorganize into a denser but less elastic gel structure. Consequently, the typical quality deterioration associated with frozen storage was manifested as increased hardness and chewiness, accompanied by decreased springiness, cohesiveness, and resilience. This trend is different from the continuous improvement effect of mixed fermentation in Ran et al. [25], indicating that excessive TSP in long-term freezing conditions may induce “hardening but loose” texture deterioration, weakening the reversible deformation ability of the network. Overall, TSP can regulate the texture of steamed buns by combining water and forming a composite network, but its freezing tolerance is inferior to that of the acid dough-lactic acid bacteria co-fermentation system. In practical applications, the amount of TSP needs to be optimized to avoid excessive damage to the texture integrity caused by long-term freezing.

### 2.5. Color of Steamed Buns

The color parameters L*, a*, and b* objectively characterize the appearance quality and browning degree of steamed buns. Specifically, L* represents lightness, a* represents the red-green tendency, and b* reflects the yellow-blue tendency. In general, steamed buns with a bright and white appearance are considered to have higher market acceptability [26].

As shown in Table 3, significant differences in color were observed between the crust and crumb of steamed buns, and these parameters were jointly influenced by frozen storage duration and TSP addition. Overall, the crumb exhibited higher L* values and lower a* values than the crust, indicating that the interior was brighter and less reddish. In contrast, the crust tended to exhibit slight color darkening and pigment enrichment, characterized by decreased L* and relatively increased a* and b* values. With increasing frozen storage time, the crust color was more sensitive to change. In several treatments, the crust b* value (yellowness) increased significantly after 30 d of frozen storage, indicating that freezing promoted surface yellowing of the final product. In contrast, the b* value of the crumb fluctuated only slightly, suggesting that the internal color was less affected by frozen storage. At the same freezing duration, TSP exhibited a certain color-stabilizing effect. Samples with higher TSP levels, particularly the 4% group, maintained relatively higher crust L* values and smaller increases in b*, resulting in a brighter appearance and reduced yellowing. The differences between crust and crumb mainly resulted from uneven heat and mass transfer during steaming. Water evaporation occurred more readily at the crust, and the resulting reduction in moisture allowed local temperatures to rise, promoting non-enzymatic browning reactions and leading to decreased L* and increased b*. In contrast, the crumb retained higher moisture and experienced more limited temperature elevation, thereby maintaining a color closer to that of the raw material [27]. Prolonged frozen storage promoted ice crystal formation and recrystallization, which disrupted the dough network and enhanced moisture migration. After thawing, the redistribution of free water and solutes facilitated surface yellowing or slight non-enzymatic browning during steaming, leading to increased b* and decreased L* values. This observation is consistent with previous reports indicating that longer frozen storage leads to darker surface colors of final products [28]. The effect of TSP can be interpreted as a combined mechanism of “water binding and structural stabilization.” Its hydrophilic groups enhanced water binding through hydrogen bonding, increased system viscosity, and formed composite networks with starch and proteins. These effects reduced moisture migration and thawing drip during frozen storage, alleviated surface solute enrichment and dehydration, and maintained uniform light scattering within the matrix. Consequently, higher L* values and suppressed increases in b* were observed. Previous studies have also demonstrated that TSP can effectively mitigate ice-crystal-induced damage and quality deterioration in frozen dough systems. Overall, steamed buns exhibited spatial color differences characterized by a brighter crumb and a more yellow-prone crust. Prolonged frozen storage mainly intensified crust yellowing (increased b* and decreased L*), whereas TSP addition partially suppressed freezing-induced moisture migration and surface solute accumulation, thereby improving crust brightness and reducing yellowing.

### 2.6. In Vitro Digestibility Characteristics of Steamed Buns

The digestive properties of steamed buns prepared from frozen dough with different frozen storage durations and TSP addition levels were evaluated through in vitro digestion assays. As shown in Table 4, at 0 d of frozen storage, the control sample (0% TSP) exhibited the highest rapidly digestible starch (RDS) content, whereas RDS gradually decreased with increasing TSP addition and reached the lowest value in the 4% group. Correspondingly, resistant starch (RS) increased significantly from 15.96% to approximately 24%, indicating that TSP promoted the formation of resistant starch. After 30 d of frozen storage, the RDS values of all groups further decreased, whereas RS increased compared with those at 0 d, while slowly digestible starch (SDS) remained relatively stable. After 60 d of frozen storage, RDS continued to decline and RS remained at a relatively higher level. Overall, longer frozen storage and higher TSP addition resulted in lower RDS and higher RS contents. This change was more pronounced during the early freezing stage (0–30 d) and tended to level off during prolonged storage (30–60 d), indicating that the major transformation occurred during the initial structural rearrangement induced by freezing [29].

The decrease in RDS and increase in RS during frozen storage can be mainly attributed to moisture migration and enhanced structural ordering. Ice crystal formation and recrystallization caused freeze concentration and local water redistribution. After thawing and steaming, these conditions facilitated starch chain reassociation and retrogradation, leading to the formation of more stable double-helix and crystalline structures typically associated with RS3. These structural changes reduced the initial hydrolysis rate and increased the RS proportion. In addition, the densification of the dough and final product structure during frozen storage restricted enzyme diffusion and substrate accessibility, causing part of the originally rapidly digestible starch to shift toward slowly digestible or resistant fractions [30]. As a hydrophilic polysaccharide, TSP enhanced water binding through hydrogen bonding and increased system viscosity, thereby reducing the diffusion and mass transfer efficiency of amylolytic enzymes within the food matrix and decreasing the exposed surface area of gelatinized starch. Moreover, polysaccharides could interact with starch or proteins to form composite networks or continuous matrix barriers at the microstructural level, further limiting enzyme accessibility and promoting the formation of more stable retrograded structures during storage. Consequently, a decrease in RDS and an increase in RS were observed with increasing TSP levels [31]. Moreover, polysaccharides could interact with starch or proteins to form composite networks or continuous matrix barriers at the microstructural level, further limiting enzyme accessibility and promoting the formation of more stable retrograded structures during storage. The above changes have clear implications for blood sugar regulation. The reduction in RDS and the increase in RS typically indicate slower postprandial glucose levels, and the expected glycemic index (eGI) decreases. Therefore, extending the freezing storage time or increasing the amount of TSP can promote the formation of resistant starch, thereby endowing steamed buns with lower blood sugar release properties.

## 3. Conclusions

This study systematically evaluated the effects of TSP on the structure, moisture distribution, quality attributes, and in vitro starch digestibility of steamed buns prepared from dough subjected to different frozen storage periods. Frozen storage impaired structural stability and physicochemical properties, as reflected by changes in crumb structure, water distribution, and texture. TSP alleviated these adverse effects and improved quality retention during prolonged storage. Specifically, TSP increased pore density and porosity, reduced average pore area, and helped maintain a more uniform pore network and higher specific volume. TSP also regulated water mobility by increasing bound water and reducing mobile water, thereby mitigating hardening and preserving textural quality. In addition, TSP partially suppressed surface yellowing and maintained higher brightness. In Vitro digestion results showed reduced rapidly digestible starch and increased resistant starch, indicating improved nutritional characteristics. Overall, TSP enhanced the quality stability and nutritional value of frozen steamed buns by stabilizing structure, regulating moisture distribution, and modulating starch digestibility. These findings support its potential as a functional ingredient in frozen wheat-based products.

## 4. Materials and Methods

### 4.1. Materials

High-gluten wheat flour (Chen Keming brand, 13.5% protein) and tamarind seed polysaccharide (TSP, purity > 90.1%, protein 1.1%, moisture 8.8%) were obtained from Youpinhui Life Supermarket (Kunming, China) and Yunnan Maoduoli (Yunnan, China), respectively. α-Amylase (porcine pancreas), amyloglucosidase, a reducing sugar kit, and a starch content kit were purchased from Shanghai Yuanye Biotechnology (Shanghai Chian), Shanghai McLean Biochemical Reagent (Shanghai Chian), Nanjing Jiancheng Science & Technology (Nanjing China), and Solarbio (Beijing, China), respectively.

### 4.2. Preparation of Steamed Buns

Wheat flour (100 g), sugar (4 g), salt (1.2 g), yeast (1.2 g), oil (4 g), and water (60 g) were mixed in a dough mixer (HKM200, Panasonic, Tokyo, Japan). The ingredients were mixed at low speed for 5 min and then at high speed for 10 min. TSP was added at levels of 0, 0.5, 1, 2, and 4 g per 100 g of flour, with 0% TSP used as the control. Store the dough at −40 °C for 0, 30 or 60 days. The frozen dough was removed from storage, thawed at 25 °C for 60 min, and proofed in a proofing box at 37 °C for 50 min. The proofed dough was then placed in a steamer and steamed for 15 min using an electric stove (1200 W). After steaming, the samples were cooled for 5 min and then removed.

The frozen storage temperature of −40 °C used in this study should be considered as a limitation. This temperature was selected because of the limitations of the available laboratory equipment, which did not allow storage at typical commercial frozen-dough temperatures. Although −40 °C is lower than conventional commercial freezing conditions, it was effective in maintaining dough structure during long-term storage. Therefore, the observed changes in pore structure, texture properties, and starch digestibility are expected to reflect qualitative trends that may also occur under commercial freezing conditions. However, further studies using conventional commercial storage temperatures are needed to confirm the applicability of these findings to industrial frozen-dough systems.

### 4.3. Determination of Pore Structure in Steamed Buns

The central slice of each steamed bun was scanned, and a 3 × 3 cm^2^ region was analyzed using ImageJ software (version 1.54p). Pore density (pores/cm^2^), average pore area (mm^2^), and porosity (%) were quantified [32].

### 4.4. Determination of Specific Volume of Steamed Buns

The specific volume of steamed buns was determined using the millet displacement method according to [25]. Each sample was measured in triplicate, and the average value was used for calculation.

Specific volume = V/m(1)

where V represents the volume of the steamed bun (mL) and m represents the mass of the steamed bun (g).

### 4.5. Moisture Migration Measurement in Steamed Bun

Moisture migration was assessed using low-field nuclear magnetic resonance (LF-NMR) with a Carr–Purcell–Meiboom–Gill (CPMG) sequence [33]. Steamed bun cubes (2 × 2 × 2 cm^3^) were placed in NMR tubes. The parameters were set as follows: echo time, 0.2 ms; number of echoes, 8000; number of accumulations, 16; and sampling interval, 3000 ms.

### 4.6. Determination of Textural Properties of Steamed Buns

The TPA test was conducted on central crumb sections with a thickness of 10 mm using a texture analyzer (TA.XT-Plus, Stable Micro Systems Ltd., Anhui, China). A P/36R probe was used under the following conditions: pre-test speed, 2.00 mm/s; test speed, 1.00 mm/s; post-test speed, 10.00 mm/s; contact force, 5 g; and compression strain, 50%. The texture parameters evaluated included hardness, springiness, adhesiveness, and chewiness [34].

### 4.7. Determination of Steamed Bun Color

The color of the steamed bun crust, measured at the central part of the top surface, and the crumb, measured at the central part of the slice, was determined using a colorimeter equipped with a C light source (Agera, HunterLab, Reston, VA, USA). The results were obtained after calibration. The parameters L* (lightness), a* (redness-greenness), and b* (yellowness-blueness) were recorded [35,36].

### 4.8. In Vitro Digestibility of Steamed Buns

Freeze-dried and ground samples passing through a 100-mesh sieve were mixed with 15 mL phosphate buffer (pH 5.2) and glass beads. The mixtures were shaken at 37 °C for 10 min and then incubated with 5 mL of enzyme mixture containing porcine pancreatic α-amylase (260 U/mL) and glucoamylase (9 U/mL). Aliquots were collected at 20 and 120 min and centrifuged at 5000× *g* for 10 min. Glucose in the supernatant was measured using a reducing sugar kit, and the total starch content was determined using a starch assay kit, following a modified method from Hudson [37].

### 4.9. Statistical Analysis

All samples were analyzed in three independent replicates. Data were expressed as mean ± standard deviation. Statistical analysis was performed using SPSS (IBM SPSS Statistics 26.0) and Origin software (Version 2024, OriginLab, Northampton, MA, USA). Two-factor analysis was performed using Duncan’s multiple range test, with the significance threshold set at *p* < 0.05.

## Figures and Tables

**Table 1:** Effects of TSP addition and frozen storage time on water distribution of *steamed buns* prepared from frozen dough.

Frozen Storage Time (Days)	Sample	A21 (%)	A22 (%)	A23 (%)
0 days	0%-TSP	20.67 ± 0.25 ^ab^	78.60 ± 0.46 ^a^	0.70 ± 0.20 ^a^
	0.5%-TSP	21.03 ± 0.06 ^a^	77.50 ± 0.10 ^b^	1.47 ± 0.15 ^b^
	1%-TSP	20.80 ± 0.10 ^ab^	77.97 ± 0.06 ^ab^	1.20 ± 0.10 ^b^
	2%-TSP	20.50 ± 0.17 ^b^	77.97 ± 0.15 ^ab^	1.50 ± 0.20 ^b^
	4%-TSP	19.50 ± 0.10 ^c^	78.57 ± 0.21 ^a^	2.20 ± 0.10 ^a^
F values		14.599	6.144	64.678
*p* values		0.000	0.009	0.000
30 days	0%-TSP	16.57 ± 0.06 ^d^	81.30 ± 0.20 ^a^	2.10 ± 0.10 ^a^
	0.5%-TSP	17.83 ± 0.15 ^b^	80.50 ± 0.10 ^b^	1.67 ± 0.15 ^b^
	1%-TSP	17.93 ± 0.06 ^b^	80.17 ± 0.15 ^c^	1.90 ± 0.10 ^ab^
	2%-TSP	17.37 ± 0.06 ^c^	80.70 ± 0.10 ^b^	1.90 ± 0.10 ^ab^
	4%-TSP	18.43 ± 0.06 ^a^	79.50 ± 0.02 ^d^	2.07 ± 0.06 ^a^
F values		11.056	3.223	19.463
*p* values		0.001	0.061	0.000
60 days	0%-TSP	19.63 ± 0.06 ^d^	78.57 ± 0.75 ^ab^	1.47 ± 0.32 ^a^
	0.5%-TSP	20.67 ± 0.06 ^a^	78.43 ± 0.25 ^b^	0.90 ± 0.20 ^ab^
	1%-TSP	20.27 ± 0.12 ^b^	78.80 ± 0.30 ^ab^	0.90 ± 0.17 ^b^
	2%-TSP	20.03 ± 0.06 ^c^	79.17 ± 0.23 ^ab^	0.77 ± 0.21 ^ab^
	4%-TSP	19.63 ± 0.06 ^d^	79.83 ± 0.75 ^a^	0.93 ± 0.31 ^a^
F values		6.077	13.698	17.171
*p* values		0.010	0.000	0.000

The concentrations labeled as 0%, 0.5%, 1%, 2%, and 4% correspond to 0, 0.5, 1, 2, and 4 g/100 g (*w*/*w*, based on wheat flour), respectively. A21: strongly bound water ratio, corresponding to water tightly associated with macromolecules; A22: weakly bound or immobilized/non-flowable water ratio, corresponding to water retained within the starch-protein network; A23: free water ratio, corresponding to mobile water located in pores or capillary spaces. Values were means ± SD (*n* ≥ 3); Means with different letters in a column were significantly different (*p* < 0.05).

**Table 2:** Effects of TSP addition and frozen storage time on textural properties of *steamed buns* prepared from frozen dough.

Frozen Storage Time (Days)	Sample	Hardness (g)	Elasticity	Cohesion	Chewing Property (g)	Recovery Property
0 days	0%-TSP	1173.25 ± 67.50 ^b^	0.86 ± 0.03 ^ab^	0.75 ± 0.07 ^a^	770.36 ± 28.8 ^a^	0.35 ± 0.02 ^a^
	0.5%-TSP	1246.59 ± 117.82 ^ab^	0.91 ± 0.02 ^a^	0.71 ± 0.05 ^a^	760.41 ± 50.82 ^a^	0.33 ± 0.02 ^ab^
	1%-TSP	1383.45 ± 93.76 ^ab^	0.84 ± 0.04 ^b^	0.67 ± 0.05 ^a^	768.81 ± 28.70 ^a^	0.28 ± 0.01 ^b^
	2%-TSP	1355.99 ± 48.98 ^ab^	0.85 ± 0.01 ^ab^	0.69 ± 0.08 ^a^	818.15 ± 63.13 ^a^	0.28 ± 0.03 ^b^
	4%-TSP	1457.04 ± 112.47 ^a^	0.86 ± 0.04 ^ab^	0.74 ± 0.07 ^a^	814.20 ± 24.60 ^a^	0.27 ± 0.03 ^b^
F values		4.512	4.012	0.763	1.284	6.781
*p* values		0.024	0.034	0.573	0.339	0.007
30 days	0%-TSP	1226.98 ± 55.82 ^b^	0.9 ± 0.02 ^a^	0.78 ± 0.04 ^a^	885.64 ± 80.48 ^a^	0.36 ± 0.03 ^a^
	0.5%-TSP	1354.78 ± 42.53 ^a^	0.86 ± 0.01 ^ab^	0.73 ± 0.01 ^a^	860.00 ± 56.00 ^ab^	0.31 ± 0.02 ^ab^
	1%-TSP	1428.62 ± 17.38 ^a^	0.86 ± 0.002 ^b^	0.69 ± 0.02 ^a^	701.95 ± 27.53 ^bc^	0.32 ± 0.01 ^ab^
	2%-TSP	1424.54 ± 55.33 ^a^	0.85 ± 0.01 ^b^	0.69 ± 0.07 ^a^	713.53 ± 45.62 ^bc^	0.3 ± 0.02 ^b^
	4%-TSP	1441.20 ± 37.17 ^a^	0.86 ± 0.02 ^b^	0.69 ± 0.04 ^a^	672.10 ± 76.39 ^c^	0.29 ± 0.01 ^b^
F values		12.422	7.329	2.701	7.961	6.106
*p* values		0.001	0.005	0.092	0.004	0.009
60 days	0%-TSP	2594.4 ± 25.02 ^b^	0.87 ± 0.02 ^a^	0.78 ± 0.03 ^a^	1707.51 ± 38.90 ^a^	0.36 ± 0.02 ^a^
	0.5%-TSP	2967.1 ± 189.77 ^ab^	0.83 ± 0.01 ^b^	0.67 ± 0.05 ^b^	1649.27 ± 83.98 ^ab^	0.29 ± 0.02 ^b^
	1%-TSP	2924.14 ± 315.8 ^ab^	0.80 ± 0.01 ^bc^	0.68 ± 0.03 ^ab^	1511.95 ± 83.67 ^bc^	0.28 ± 0.02 ^bc^
	2%-TSP	3242.1 ± 338.86 ^a^	0.81 ± 0.02 ^b^	0.64 ± 0.05 ^b^	1669.96 ± 37.93 ^ab^	0.27 ± 0.03 ^bc^
	4%-TSP	3476.58 ± 108.29 ^a^	0.77 ± 0.01 ^c^	0.62 ± 0.02 ^b^	1408.73 ± 51.95 ^c^	0.23 ± 0.01 ^c^
F values		6.400	20.344	7.823	11.925	13.421
*p* values		0.008	0.000	0.004	0.001	0.000

The concentrations labeled as 0%, 0.5%, 1%, 2%, and 4% correspond to 0, 0.5, 1, 2, and 4 g/100 g (*w*/*w*, based on wheat flour), respectively. Values were means ± SD (*n* ≥ 3); Means with different letters in a column were significantly different (*p* < 0.05).

**Table 3:** Effects of TSP addition and frozen storage time on the color of *steamed buns* prepared from frozen dough.

Frozen Storage Time (Days)	Sample	Crust Color			Crumb Color		
		L*	a*	b*	L*	a*	b*
0 days	0%-TSP	75.25 ± 0.75 ^b^	0.12 ± 0.05 ^c^	20.25 ± 0.46 ^a^	79.72 ± 0.62 ^ab^	0.20 ± 0.02 ^a^	18.05 ± 0.02 a
	0.5%-TSP	75.46 ± 0.59 ^b^	0.23 ± 0.06 ^bc^	19.11 ± 0.26 ^b^	77.38 ± 1.46 ^b^	0.22 ± 0.07 ^a^	16.85 ± 0.15 b
	1%-TSP	76.48 ± 1.10 ^ab^	0.31 ± 0.04 ^ab^	18.26 ± 0.52 ^bc^	79.92 ± 0.73 ^b^	0.12 ± 0.05 ^ab^	18.17 ± 0.42 a
	2%-TSP	77.41 ± 0.13 ^a^	0.42 ± 0.03 ^a^	18.19 ± 0.27 ^bc^	79.42 ± 0.91 ^ab^	0.04 ± 0.02 ^b^	18.25 ± 0.38 a
	4%-TSP	78.07 ± 0.08 ^a^	0.30 ± 0.04 ^ab^	18.03 ± 0.35 ^c^	80.61 ± 0.50 ^a^	0.13 ± 0.03 ^ab^	17.62 ± 0.36 ab
F values		10.348	18.927	17.673	5.399	8.712	10.584
*p* values		0.001	0.000	0.000	0.014	0.003	0.001
30 days	0%-TSP	74.61 ± 1.05 ^b^	0.38 ± 0.06 ^a^	22.94 ± 0.14 ^b^	80.31 ± 0.25 ^a^	0.18 ± 0.03 ^a^	19.70 ± 0.22 a
	0.5%-TSP	75.39 ± 1.40 ^ab^	0.36 ± 0.08 ^a^	22.43 ± 0.68 ^b^	78.84 ± 0.64 ^a^	0.07 ± 0.05 ^c^	20.33 ± 0.54 a
	1%-TSP	73.43 ± 0.14 ^b^	0.37 ± 0.02 ^a^	25.69 ± 0.67 ^a^	78.94 ± 1.36 ^a^	0.08 ± 0.04 ^bc^	20.03 ± 0.32 a
	2%-TSP	74.73 ± 0.75 ^b^	0.40 ± 0.08 ^a^	22.75 ± 0.51 ^b^	79.99 ± 0.01 ^a^	0.06 ± 0.02 ^c^	18.41 ± 0.03 b
	4%-TSP	77.02 ± 0.51 ^a^	0.06 ± 0.04 ^b^	19.77 ± 0.17 ^c^	79.97 ± 0.26 ^a^	0.16 ± 0.01 ^ab^	19.57 ± 0.02 a
F values		8.998	16.743	54.240	2.814	10.304	18.024
*p* values		0.002	0.000	0.000	0.084	0.001	0.000
60 days	0%-TSP	74.95 ± 0.12 ^b^	0.56 ± 0.09 ^ab^	18.79 ± 0.41 ^b^	78.64 ± 0.95 ^b^	0.44 ± 0.04 ^ab^	18.97 ± 0.21 ab
	0.5%-TSP	76.83 ± 0.06 ^a^	0.47 ± 0.01 ^b^	19.02 ± 0.12 ^a^	79.14 ± 0.15 ^b^	0.30 ± 0.01 ^c^	19.05 ± 0.32 a
	1%-TSP	75.83 ± 0.73 ^ab^	0.22 ± 0.02 ^c^	19.53 ± 0.18 ^ab^	78.36 ± 0.50 ^b^	0.52 ± 0.09 ^a^	17.59 ± 1.06 b
	2%-TSP	74.79 ± 0.16 ^b^	0.66 ± 0.09 ^a^	18.46 ± 0.36 ^b^	78.46 ± 0.46 ^b^	0.34 ± 0.03 bc	19.38 ± 0.18 a
	4%-TSP	76.99 ± 0.29 ^a^	0.6 ± 0.05 ^ab^	19.96 ± 0.40 ^a^	80.67 ± 0.43 ^a^	0.39 ± 0.04 ^abc^	18.15 ± 0.20 ab
F values		6.834	26.212	10.755	8.590	8.454	6.015
*p* values		0.006	0.000	0.001	0.003	0.003	0.010

The concentrations labeled as 0%, 0.5%, 1%, 2%, and 4% correspond to 0, 0.5, 1, 2, and 4 g/100 g (*w*/*w*, based on wheat flour), respectively. L*: Brightness (0 = black, 100 = white); a*: Red-green coloration (positive value indicates red, negative value indicates green); b*: Yellow-blue coloration (positive value indicates yellow, negative value indicates blue). Values were means ± SD (*n* ≥ 3); Means with different letters in a column were significantly different (*p* < 0).

**Table 4:** Effects of TSP addition and frozen storage time on in vitro digestive properties of *steamed buns* prepared from frozen dough.

Frozen Storage Time (Days)	Sample	RDS (%)	SDS (%)	RS (%)
0 days	0%-TSP	50.65 ± 1.61 ^a^	34.48 ± 1.28 ^a^	15.96 ± 0.84 ^c^
	0.5%-TSP	47.47 ± 0.58 ^ab^	32.94 ± 0.35 ^ab^	20.50 ± 1.02 ^b^
	1%-TSP	48.52 ± 0.92 ^b^	29.99 ± 1.69 ^b^	22.42 ± 0.53 ^bc^
	2%-TSP	44.85 ± 1.54 ^bc^	33.16 ± 0.72 ^ab^	24.50 ± 0.52 ^a^
	4%-TSP	43.66 ± 1.40 ^c^	34.29 ± 1.67 ^a^	24.27 ± 0.75 ^ab^
F values		14.599	6.144	64.678
*p* values		0.000	0.009	0.000
30 days	0%-TSP	47.08 ± 0.88 ^a^	37.05 ± 1.33 ^a^	17.52 ± 0.57 ^c^
	0.5%-TSP	43.60 ± 0.94 ^b^	36.34 ± 0.76 ^ab^	20.72 ± 0.83 ^b^
	1%-TSP	43.38 ± 0.46 ^b^	36.6 ± 0.55 ^ab^	19.62 ± 1.23 ^bc^
	2%-TSP	43.62 ± 1.08 ^b^	34.52 ± 0.99 ^b^	21.60 ± 0.62 ^ab^
	4%-TSP	42.72 ± 0.98 ^b^	35.97 ± 0.83 ^ab^	23.00 ± 0.64 ^a^
F values		11.056	3.223	19.463
*p* values		0.001	0.061	0.000
60 days	0%-TSP	45.24 ± 0.75 ^a^	36.60 ± 0.63 ^ab^	17.76 ± 0.86 ^b^
	0.5%-TSP	42.25 ± 1.42 ^b^	37.52 ± 0.86 ^a^	19.65 ± 0.70 ^b^
	1%-TSP	42.12 ± 0.95 ^b^	34.09 ± 0.67 ^c^	23.36 ± 0.83 ^a^
	2%-TSP	42.70 ± 1.12 ^ab^	35.56 ± 0.63 ^bc^	22.41 ± 1.01 ^a^
	4%-TSP	41.28 ± 0.90 ^b^	37.23 ± 0.93 ^a^	22.43 ± 1.36 ^a^
F values		6.077	13.698	17.171
*p* values		0.010	0.000	0.000

The concentrations labeled as 0%, 0.5%, 1%, 2%, and 4% correspond to 0, 0.5, 1, 2, and 4 g/100 g (*w*/*w*, based on wheat flour), respectively. RDS: Rapidly digested starch; SDS: Slowly digested starch; RS: Resistant starch. Values were means ± SD (*n* ≥ 3); Means with different letters in a column were significantly different (*p* < 0.05).

## Data Availability

The original contributions presented in this study are included in the article. Further inquiries can be directed to the corresponding author.
